# An analysis of time trends in breast and prostate cancer mortality rates in Lithuania, 1986–2020

**DOI:** 10.1186/s12889-022-14207-4

**Published:** 2022-09-23

**Authors:** Rūta Everatt, Daiva Gudavičienė

**Affiliations:** 1 Laboratory of Cancer Epidemiology, National Cancer Institute, Baublio 3B, LT-08406 Vilnius, Lithuania; 2 Department of Plastic and Reconstructive Surgery, Vilnius University Hospital Santaros Klinikos, Vilnius, Lithuania; 3 Breast Surgery and Oncology Department, National Cancer Institute, Vilnius, Lithuania

**Keywords:** Breast cancer, Prostate cancer, Mortality, Trends, Screening, Lithuania

## Abstract

**Background:**

Breast cancer (BC) and prostate cancer (PC) mortality rates in Lithuania remain comparatively high despite the ongoing BC and PC screening programmes established in 2006. The aim of this study was to investigate time trends in BC and PC mortality rates in Lithuania evaluating the effects of age, calendar period of death, and birth-cohort over a 35-year time span.

**Methods:**

We obtained death certification data for BC in women and PC in men for Lithuania during the period 1986–2020 from the World Health Organisation database. Age-standardised mortality rates were analysed using Joinpoint regression. Age-period-cohort models were used to assess the independent age, period and cohort effects on the observed mortality trends.

**Results:**

Joinpoint regression analysis indicated that BC mortality increased by 1.6% annually until 1996, and decreased by − 1.2% annually thereafter. The age-period-cohort analysis suggests that temporal trends in BC mortality rates could be attributed mainly to cohort effects. The cohort effect curvature showed the risk of BC death increased in women born prior to 1921, remained stable in cohorts born around 1921–1951 then decreased; however, trend reversed in more recent generations. The period effect curvature displayed a continuous decrease in BC mortality since 1991–1995. For PC mortality, after a sharp increase by 3.0%, rates declined from 2007 by − 1.7% annually. The period effect was predominant in PC mortality, the curvature displaying a sharp increase until 2001–2005, then decrease.

**Conclusions:**

Modestly declining recent trends in BC and PC mortality are consistent with the introduction of widespread mammography and PSA testing, respectively, lagging up to 10 years. The study did not show that screening programme introduction played a key role in BC mortality trends in Lithuania. Screening may have contributed to favourable recent changes in PC mortality rates in Lithuania, however the effect was moderate and limited to age groups < 65 years. Further improvements in early detection methods followed by timely appropriate treatment are essential for decreasing mortality from BC and PC.

**Supplementary Information:**

The online version contains supplementary material available at 10.1186/s12889-022-14207-4.

## Background

Breast cancer (BC) is the leading tumour in terms of incidence and the most common cause of cancer death among women in Europe and in Lithuania [1]. Prostate cancer (PC) is the most common cancer diagnosis in men in most high-income countries and in Lithuania; it is the second most common cause of cancer death [1]. BC and PC mortality trends were declining in recent years in many countries, reductions were associated mainly with the combined effects of earlier detection and improved awareness and treatment [2–4]. Effective organized population-based BC screening programmes, implemented in many Northern and Western European countries in the late 1980s, have been related to the reduced BC mortality; whereas the role of extensive opportunistic prostate-specific antigen (PSA)-based testing for PC remains uncertain [1, 2, 4–9]. In Central and Eastern Europe, modest and late decreases or the continued increase in BC and PC mortality was observed; unfavourable trends remain largely unexplained and are only partly attributable to less accessible or delayed modern effective treatment [1–3, 5, 9–11]. Similar epidemiological features have been shown between BC and PC, implying common causal pathways, including hormonal, metabolic, genetic, dietary and other factors [6, 7, 12].

The BC incidence rates in Lithuania are lower, but the mortality rates are higher compared to most Northern and Western European countries [1, 9]. The national population-based BC prevention programme in Lithuania was started in October, 2005, fully implemented in 2006, targeting women aged 50–69 years at two-year intervals [13]. However, the programme is lacking all the necessary elements of organized population-based screening, including written invitation with prefixed appointment for all eligible women, screening registry and appropriate systematic quality assurance, whereas the examination coverage is low (45% in 2014) [14].

In Western and Northern European countries, although PC incidence trends increased, mortality rates have been declining since the 1990s [6, 7, 15]. In Central and Eastern Europe declines in mortality trends started later and were less pronounced [1, 3, 10, 16]. It has been shown that repeated PC screening using PSA testing reduces PC mortality risk by 20% [17]. However, population PSA testing is considered controversial due to potential overdiagnosis and overtreatment of clinically insignificant PC [17–19]. There are substantial differences in recommendations by national and international professional associations, European Union and the European Code Against Cancer [19–24]. In Lithuania, PSA test was introduced into clinical practice in 2000, and a nationwide PC screening programme was started in 2006, targeting all men aged 50–75 years and 45–49 years with family history of PC, annually. Biennial PC screening from 2009 and target age 50–69 years from 2017 were introduced. Similar to other screening programmes in Lithuania, screening registry, systematic written invitation or appropriate screening quality assurance are lacking [25, 26]. Although Lithuania is the only country in the world with an implemented PSA-based systematic PC screening [24], the age-standardized PC mortality rate (ASMR) was 3rd highest and 4th highest in Europe in 2015–2018 and in 2020, respectively [3, 9].

Despite the high burden of both tumours in Lithuania, no evaluation of age, period and cohort effects on mortality trends has been performed. The aim of this study was to assess and interpret time trends in BC and PC mortality in Lithuania with particular focus on independent effects of age, time period and birth-cohort in order to better understand the possible impact of screening practices.

## Methods

We extracted official data for deaths of BC and PC in Lithuania for the period 1986–2020 from the World Health Organisation (WHO) mortality database [27]. The 2020 was the last available year for Lithuania in the WHO database. Population counts for each calendar year by sex and 5-year age categories were obtained from the official Statistics Lithuania portal [28].

Joinpoint regression was used to analyse trends in age-standardised mortality rates (ASMR) (world standard population) per 100,000 for BC and PC for the years 1986–2020. We depicted annual ASMRs for each tumour. The time points called ‘joinpoints’ were identified when a change in the linear slope of the temporal trend occurred [29]. A maximum number of three Joinpoints was allowed. The estimated annual percent change (APC) was computed for each identified linear segment. The age-specific mortality rates across the 5-year time periods were calculated as the number of new patients per 100,000 person-years, using 5-year age groups (BC 25–29 to 85+ years; PC 45–49 to 85+ years).

With the aim of a more detailed analysis, the age, period and cohort effects were calculated using an age-period-cohort analysis Web tool (http://analysistools.nci.nih.gov/apc/) [30]. For this purpose, data were grouped by 5-year age and period intervals, excluding those aged < 25 years for BC analysis and < 45 years for PC analysis due to small number of deaths in these groups. Using the Web tool, we obtained: longitudinal age-specific rates (i.e. fitted age-specific rates in reference cohort adjusted for period deviations), period rate ratios (RRs) and cohort RRs. We used 2006–2010 period (which corresponds to the introduction of screening programmes) as our reference period and the 1946 birth cohort (which is central cohort for BC) as our reference cohort. We also obtained the Net Drift, i.e. model-based estimates of an average APC in the ASMRs over the entire 35-year period; and Local drifts, i.e. age-specific APCs over time. We used the Wald Chi-Square test to determine statistical parameters in the age, period and cohort model. The Web tool is described in detail elsewhere [30]. All tests of statistical significance were two-sided, a *P* value of < 0.05 was considered statistically significant.

## Results

### Breast cancer age standardised and age-specific mortality trends

A total of 18,668 deaths from BC were reported in Lithuania from 1986 to 2020 (Table 1). The number of deaths due to BC in age group 25–49 years was 2795 deaths (15%), whereas at age ≥ 70 years - 7265 deaths (39%).

**Table 1:** Age-specific and age-standardized (world population) mortality rates^a^ and numbers of deaths (N) from breast and prostate cancer in Lithuania, by calendar period

Age at death	1986–2020	1986–1990	1991–1995	1996–2000	2001–2005	2006–2010	2011–2015	2016–2020
	N (%)	Rate (N)	Rate (N)	Rate (N)	Rate (N)	Rate (N)	Rate (N)	Rate (N)
Breast cancer								
25–29	31 (0.2)	0.5 (4)	0.9 (6)	1.1 (7)	0.3 (2)	0.6 (3)	0.8 (4)	1.1 (5)
30–34	191 (1.0)	7.4 (50)	5.0 (38)	3.7 (25)	3.2 (20)	3.3 (18)	4.7 (21)	4.3 (19)
35–39	435 (2.3)	14.9 (92)	13.9 (91)	11.6 (84)	6.8 (44)	9.1 (53)	7.2 (35)	8.7(36)
40–44	789 (4.2)	26.2 (147)	26.6 (159)	19.5 (121)	18.5 (129)	14.4 (88)	14.6 (80)	14.0 (65)
45–49	1349 (7.2)	35.9 (215)	40.1 (220)	41.4 (235)	37.3 (223)	28.0 (185)	26.0 (149)	23.4 (122)
50–54	1775 (9.5)	48.5 (290)	53.6 (312)	51.5 (268)	49.1 (266)	44.1 (250)	35.7 (223)	30.1 (166)
55–59	2205 (11.8)	56.0 (330)	61.2 (354)	64.2 (355)	63.9 (314)	58.0 (297)	54.7 (293)	43.6 (262)
60–64	2376 (12.7)	61.4 (333)	69.0 (389)	68.4 (371)	70.4 (370)	72.9 (336)	62.5 (304)	52.9 (273)
65–69	2252 (12.1)	65.2 (244)	68.7 (347)	76.0 (392)	62.1 (317)	71.1 (350)	69.0 (298)	66.2 (304)
70–74	2327 (12.5)	65.2 (184)	83.1 (276)	80.4 (356)	96.7 (450)	82.9 (386)	84.0 (377)	75.1 (298)
75–79	2065 (11.1)	67.8 (188)	72.1 (165)	88.0 (237)	101.9 (375)	95.0 (377)	90.1 (362)	91.9 (361)
80–84	1548 (8.3)	62.6 (115)	74.3 (144)	87.7 (139)	111.3 (216)	108.5 (298)	101.7 (306)	104.9 (330)
85+	1325 (7.1)	57.0 (72)	55.6 (82)	99.2 (151)	103.9 (140)	130.8 (208)	126.6 (279)	148.1 (393)
All	18,668 (100)	16.8 (2264)	18.2 (2583)	18.3 (2741)	17.7 (2866)	16.7 (2849)	15.5 (2731)	14.2 (2634)
Prostate cancer								
25–29	2 (0.01)	0 (0)	0 (0)	0.2 (1)	0 (0)	0 (0)	0.2 (1)	0 (0)
30–34	0 (0)	0 (0)	0 (0)	0 (0)	0 (0)	0 (0)	0 (0)	0 (0)
35–39	3 (0.02)	0.2 (1)	0.3 (2)	0 (0)	0 (0)	0 (0)	0 (0)	0 (0)
40–44	8 (0.05)	0.4 (2)	0.4 (2)	0.3 (2)	0.2 (1)	0.2 (1)	0 (0)	0 (0)
45–49	57 (0.4)	0.8 (4)	1.4 (7)	2.4 (12)	2.0 (11)	1.8 (11)	1.5 (8)	0.8 (4)
50–54	192 (1.3)	4.0 (20)	4.5 (22)	6.5 (28)	7.1 (33)	8.0 (39)	5.1 (28)	4.5 (22)
55–59	552 (3.7)	11.1 (51)	18.6 (85)	17.9 (78)	19.5 (76)	25.1 (103)	17.6 (77)	16.3 (82)
60–64	1124 (7.5)	29.8 (103)	42.6 (173)	41.9 (164)	44.1 (169)	58.5 (196)	41.2 (147)	43.9 (172)
65–69	1933 (12.9)	69.5 (154)	91.6 (269)	90.3 (301)	91.7 (306)	103.1 (331)	101.3 (282)	95.6 (290)
70–74	2709 (18.1)	137.3 (204)	161.9 (284)	174.4 (393)	179.3 (478)	185.3 (491)	187.5 (479)	170.2 (380)
75–79	3182 (21.3)	177.6 (253)	223.4 (235)	315.6 (384)	324.9 (519)	345.2 (668)	302.0 (589)	279.7 (534)
80–84	2832 (18.9)	255.7 (258)	318.1 (275)	391.6 (241)	507.0 (368)	529.4 (526)	503.9 (622)	423.1 (542)
85+	2369 (15.8)	267.5 (137)	334.4 (220)	459.4 (276)	593.6 (278)	756.9 (369)	702.0 (453)	754.6 (636)
All	14,963 (100)	11.1 (1187)	14.3 (1574)	16.5 (1880)	18.1 (2239)	20.5 (2735)	18.6 (2686)	17.6 (2662)

^a^ per 100,000

**Fig. 1:**
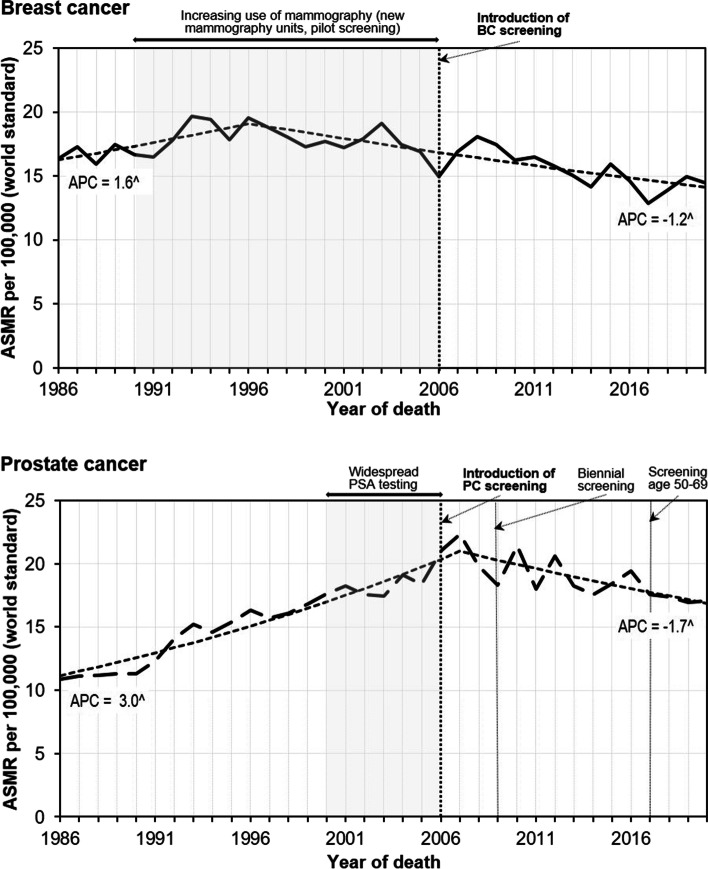
Modelled trends (dotted line) from Joinpoint regression versus the observed age-standardized mortality rates (ASMR) from breast and prostate cancer and annual percentage change (APC) in Lithuania, 1986–2020. ^ - the APC is significantly different from zero

BC mortality trend showed one joinpoint with initial modest increase to 19.5 per 100,000 in 1996 (APC = 1.6, 95% confidence interval [CI]: 0.3; 2.9), followed by a modest decline thereafter to 14.5 per 100,000 in 2020 (APC = −1.2, 95% CI: −1.6; −0.9) (Fig. 1).

**Fig. 2:**
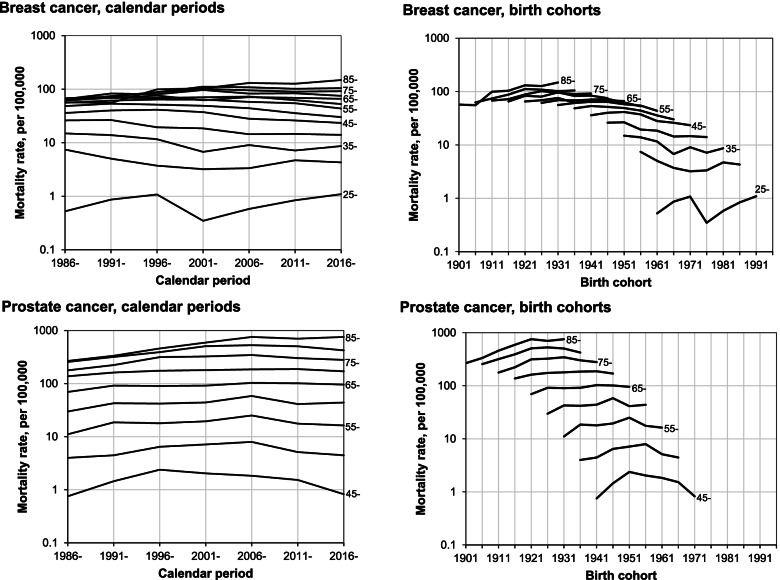
Age-specific breast and prostate cancer mortality rates by calendar period and birth cohort in Lithuania, 1986–2020

The age-specific mortality rates of BC by calendar period and birth cohort are presented in Fig. 2. Although the mortality rates did not show a clear pattern over the successive calendar periods, a decrease since approximately 1991–1995 was noticeable in the younger age groups. In BC mortality, cohort effects were more expressed than period effects. The risk of death increased, stabilized and then decreased with each subsequent cohort born up to 1966. Decline in mortality levelled off and increased in successive younger generations.

### Breast cancer mortality trends, age-period-cohort analysis

**Fig. 3:**
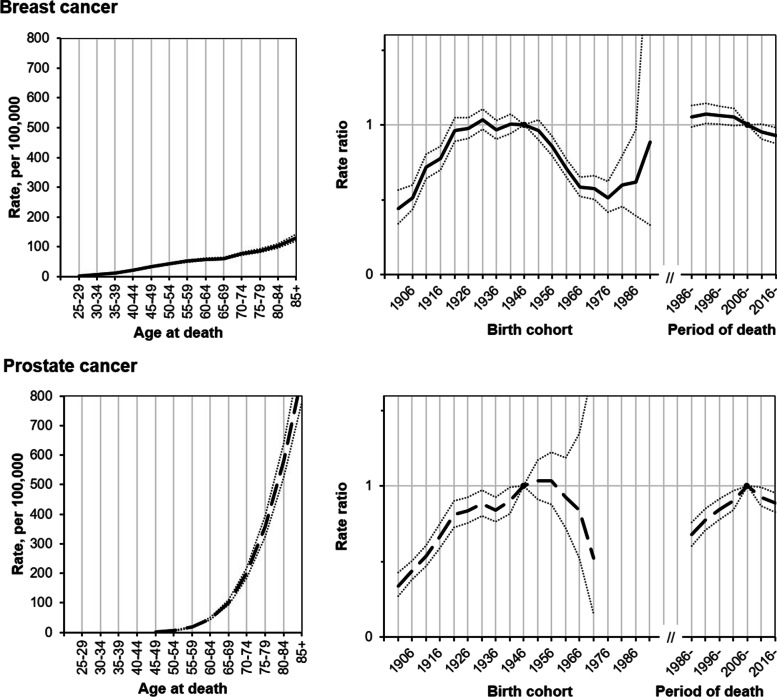
Estimated age, birth cohort, and period effects and 95% confidence intervals from age–period–cohort analysis of mortality rates of breast and prostate cancer in Lithuania, 1986–2020

Figure 3 presents the age effects and RRs for each period and cohort by cancer type, estimated in the age-period-cohort analysis. The longitudinal age curve for BC mortality displays a monotonic pattern: rates started to increase from 30–34 years of age, and gradually increased until ≥80 years of age. There was a steep rise in cohort effect among the cohorts born between 1901 and 1921, followed by levelling off and stabilization until 1946 cohort (Fig. 3, Supplementary Table A). The mortality risk for BC rapidly fell in cohorts 1951–1976, but then reversed upwards in most recent cohorts. Our analysis showed that the BC mortality risk started to decline from 1991–1995, downward trend accelerated from 2001–2005. Declining period effect during the last decade was observed: compared to 2006–2010, the RRs in 2016–2020 was 0.93 (95% CI: 0,88; 0.98).

**Fig. 4:**
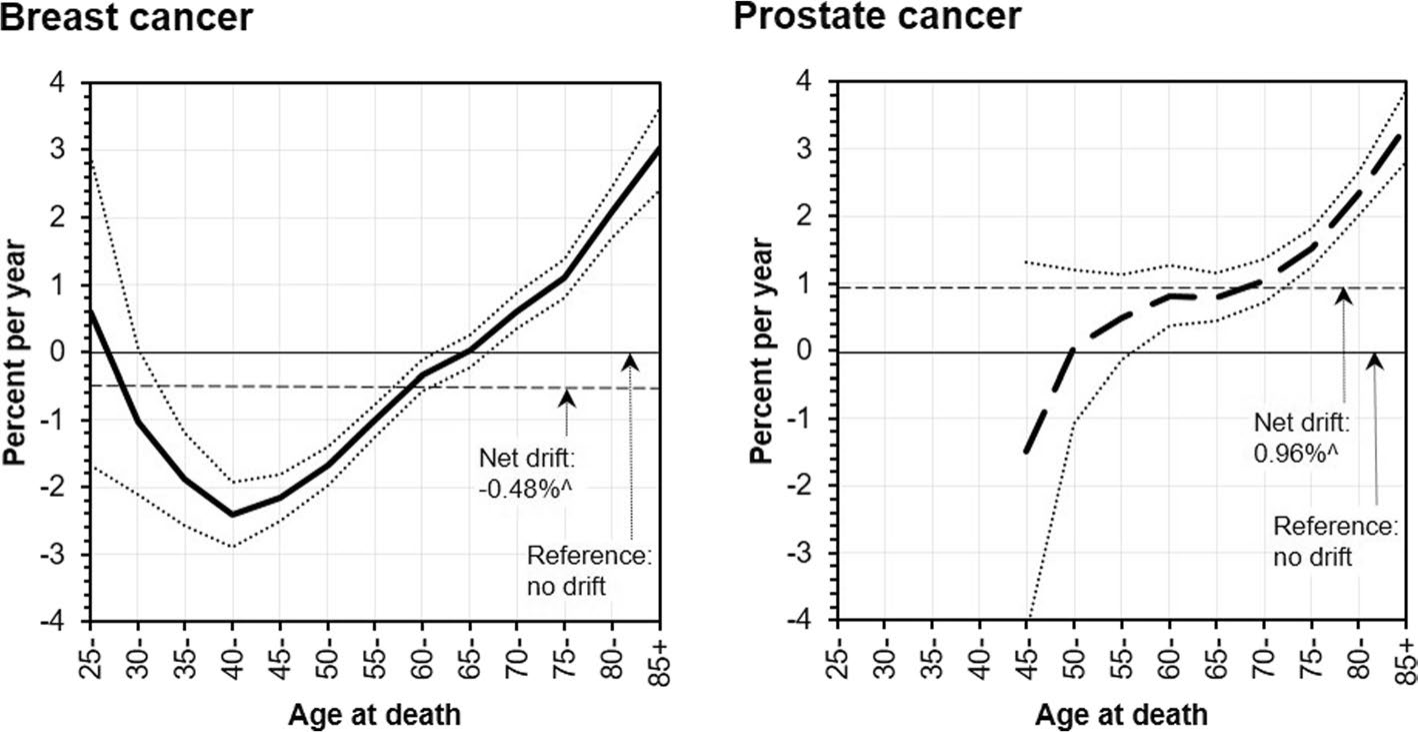
Local drift values (i.e. estimated age-specific annual percent change) in the mortality rates of breast and prostate cancer in Lithuania, 1986–2020. ^ - the APC is significantly different from zero

Wald Chi-Square tests showed statistically significant age and cohort effects in BC mortality trends (Supplementary Table B). The net drifts and local drifts are illustrated in Fig. 4. The net drifts showed small but statistically significant downward trend in BC mortality by − 0.48% (95% CI: − 0.71; − 0.26) per year. The local drifts showed an increase by 1 to 3% per year in older groups, no significant change in age groups 65 to 69 years, and a marked decrease by 1 to 2.4% per year among 30–34 to 60–64 years old age groups .

### Prostate cancer age standardised and age-specific mortality trends

A total of 14,963 PC deaths were reported in Lithuania from 1986 to 2020 (Table 1). About three quarters (74%, 11,092 deaths) of PC deaths were at age ≥ 70 years. Conversely, the number of deaths due to PC in age group 25–49 years was low (0.5%, 70 deaths). Joinpoint regression analysis showed that the PC mortality trend increased rapidly from 1986 to 2007 by 3.0% (95% CI: 2.6; 3.5) per year, then declined by − 1.7% (95% CI: − 2.4; − 0.9) per year (Fig. 1).

The analysis of age-specific mortality rates of PC by calendar period showed clear increase in rates over time until the 2006–2010 followed by downward trend in the age groups 45–64 years and no change in men aged 65 years and older (Fig. 2). The PC mortality did not show any clear pattern over the successive birth cohorts.

### Prostate cancer mortality trends, age-period-cohort analysis

Age, period and cohort effects were significant in PC mortality trends (Fig. 3, Supplementary Table B). The longitudinal age curve displays an increase in PC mortality that started from age 50–54, the association between age and mortality risk was J-shaped. There was a steep rise in cohort effect among the men born between 1901 and 1921, followed by levelling off until 1936. The mortality risk further increased in cohorts born up to 1946, then stabilized and fell (Fig. 3, Supplementary Table A). Our analysis showed the significant period effect; namely, the PC mortality risk steeply increased prior to 2006, then declined. Compared to 2006–2010, the RR in 2016–2020 was 0.89 (95% CI: 0.83; 0.96).

The net drifts and local drifts are illustrated in Fig. 4. The net drifts showed statistically significant upward trend in PC mortality by 0.96% (95% CI: 0.55; 1.37) per year during the entire study period. The local drifts showed an increase by 0.5 to 3% per year in older age groups (60 years and older), and no significant change in age groups 50 to 59 years (Fig. 4).

## Discussion

The study showed that BC age-standardized mortality rates in Lithuania increased by 1.6% annually during the period 1986–1996, then declined by 1.2% per year during 1996–2020. The age-period-cohort analysis suggests that temporal trends in BC mortality could be attributed predominantly to birth cohort effects, implicating contribution of the changes in the prevalence of BC risk factors across generations. The declining period effect in BC mortality trends suggests the beneficial effect of increased mammography testing, as well as general improvements in early detection and new treatments. In PC mortality, a pronounced 3.0% annual increase from 1986 to 2007, followed by a moderate 1.7% decline, was observed. There were differences among age groups, with more favourable trends observed in middle-aged (45–64 years) men. The predominance of period effect over birth cohort effect in PC mortality was observed suggesting the role of increased diagnostic activity using PSA testing and new treatments. An implementation of the screening programme may have contributed to favourable recent trends, particularly in men aged below 65 years.

The age-period-cohort analysis of mortality trends showed that the most prominent effect in BC was the cohort effect. The bell-shaped cohort effect pattern was similar to previous results from white populations, that were related to the combined effects of changes in reproductive factors, overweight and obesity, hormone replacement therapy and screening mammography [7, 31, 32]. It is likely that postponement of the first birth and having fewer children had an impact on increasing BC mortality risk in older cohorts in Lithuania. A steep decline in cohorts born since 1946 could not be explained by changes in BC risk factors. Similar unexplained declines were reported among European women [2, 32]. The analysis showed a change point in the cohort effect in youngest generations, born from 1976 onward, when the BC mortality risk increased. Risk factors during adolescence or early adulthood, e.g. increased prevalence of overweight or obesity, lower levels of physical activity, increased alcohol intake, contraceptive use, further changes in childbearing habits could have played a role. The prevalence of obesity among < 25 years old women in Lithuania increased from 1% in 2005 to 8% in 2019 [28]; the intake of strong alcohol ≥1 times per week increased from 4% in 1994 to 10% in 2015; the intake of beer - from 10 to 21%, respectively [33, 34]. In addition, contraceptive use among women aged 15–49 years increased from 51% in 1995 to 69% in 2009 [35].

In comparison to most European countries, where decreases since mid-1980s by at least 2% annually have been reported; in Lithuania BC mortality rates peaked later and annual reductions were smaller [2, 5–7, 36, 37]. The period effect in BC mortality trends decreased gradually since 1991–1995 in Lithuania, no period-specific effect of screening programme was detected. Notably, the BC mortality in Lithuania started to decline prior to the introduction of the screening programme, suggesting that beneficial effects could possibly be attributed to increased mammography testing, general improvements in early detection and subsequent new treatments of earlier diagnosed cases [2, 36]. The mammography was increasingly used since the beginning of 1990s, including newly installed mammography units and pilot screening programmes that possibly contributed to the sharp rise in BC incidence rates from 29.0 per 100,000 in 1990 to 41.5 per 100,000 in 2002 [38, 39], followed by a subsequent decline in BC mortality rates due to early diagnosis. In 2004, i.e. before the screening implementation, 17% of women reported having had mammography [40]. After the introduction of national screening programme, the mammography testing increased; however, the screening examination coverage remained comparatively low, 45% vs. 72–84% in Scandinavian countries or United Kingdom [14, 33]. Our study showed declines in BC mortality also in women 25–49 years of age, i.e. younger than the target age groups. This result is in agreement with previous studies and possibly reflects an increased population awareness of BC and mammography testing, also improved diagnostics and treatment of BC that impacted younger women [5, 6].

Relatively slow decline in BC mortality rates may partly be explained by the lack of timely and appropriate treatment that is required after early detection. About one-third of the decline in BC mortality in Western Europe and North America is assumed to be due to screening and better diagnosis, whereas about two-thirds – due to innovative treatment methods [2]. In order to substantially decrease BC mortality in Lithuania, further improvements in health-care system efficiency and access to effective treatment are essential, including efficient treatment regimens, multidisciplinary approach, adequate cancer services and facilities as well as access to these services [31, 37].

A pronounced increase in PC mortality was observed from 1986 to 2007 in Lithuania. The age-period-cohort analysis showed the predominant period effect in PC mortality trend, steeply increasing until 2006–2010. This finding is consistent with an increased awareness among the population and professionals and active case searching practices including intensive opportunistic PSA testing. PSA testing became widely available since 2000 in Lithuania and possibly played important role in rising PC mortality [9, 11]. Our result is in agreement with Center et al. [41], showing that the PC incidence rates in Lithuania increased from mid-1980s, with a rapid rise by 22.4% per year between 2000 and 2006, corresponding to the introduction of opportunistic PSA testing [11]. Moreover, the use of advanced diagnostic imaging and radical treatments may have contributed to the increasing detection of indolent tumours with no or weak life threatening potential and rising PC mortality rates due to misattribution of the cause of death [32, 42]. An increase in mortality rates in 80–84 and 85+ year old men suggest that diagnostic procedures were actively performed also in this age group, although the benefit was unlikely [11]. The present study observed decline in risk of death due to PC since 2006–2010, particularly among men below 65 years of age. Similar result was apparent in a recent study, which observed a decrease in PC mortality in Lithuania in 2015–2018 versus 2005–2009 for men all ages and in the age group 35–64 years [3]. This is consistent with the introduction of opportunistic PSA testing in 2000 and suggests beneficial effects of earlier diagnosis and effective early treatment in these age groups. Previous studies have shown the time lag of 7–9 years between the increasing PSA testing and subsequent reductions in mortality due to beneficial treatment of earlier diagnosed cases [6, 7]. More conservative use of PSA testing (less screening outside the target age groups, longer screening interval) may have also contributed to the reduction in misattributed cause of death and decreasing mortality rates [11, 42, 43]. Despite the implemented organized national screening programme, the favourable tendency in PC mortality in Lithuania was weak compared to European men, with the death rates remaining among the highest in Europe [3, 6, 7, 10, 32]. Furthermore, we observed the positive annual net drift of 0.96% and age-specific local drifts, showing that the mortality rates were higher in 2016–2020 compared to baseline 1986–1990. This result may possibly be explained by ineffective screening programme as well as differences in availability and access to important treatments, including surgery, hormonal and radiation therapy, compared to the more affluent countries [10, 18].

The cohort effect curvature for PC mortality showed similar pattern with BC pattern. The risk factors for PC remain mostly unidentified, however common factors like “westernization” (increasing obesity, dietary fat consumption and reduced physical activity) could probably explain similarity in cohort effects in BC and PC mortality in older generations. The interpretation of changes in 1936 to 1966 birth cohorts is complicated due to increased diagnostic activity and improved PC treatment.

Our results suggest that opportunistic PSA-based screening programme may have somewhat contributed to the downward PC mortality trend in Lithuania, but the effect was modest. The role of PSA testing in PC mortality reduction and balance between benefits and risks remains equivocal due to overdiagnosis and overtreatment [8, 41, 44, 45]. Instead of the PSA-only diagnostic strategy, new early PC detection algorithms and technologies have been suggested in order to differentiate life-threatening PC from clinically insignificant PC, using urine, serum or tissue biomarkers, risk calculators, multivariable prediction models and imaging by MRI [22–24].

The strength of our study is the comprehensive quantification and comparison of BC and PC mortality trends using the high-quality cancer mortality data from the WHO mortality database. The study has several limitations. First, interpretation of results is complicated because declining mortality rates in Lithuania could reflect either the impact of the early diagnosis using widespread testing or the improved treatment, as they occurred at a similar time period. Second, sharp changes for the youngest cohorts may be less stable and should be interpreted with caution because of few age-specific rates and small number of cancer cases; however, recent death rates in the young may carry important information for future trends.

## Conclusions

Moderate declines in mortality rates from BC and PC since around 1996 and 2007, respectively, were observed, reflecting favourable effects from widespread mammography and PSA testing after a lag up to 10 years. For BC mortality, the significant cohort effect suggests the importance of changes in risk factors. For PC mortality, the significant period effect shows the impact of improvements in early diagnostics and new treatments of PC. Although disentangling the importance of different measures as well as an impact of overdiagnosis is difficult, the study suggest that implementation of screening programme may have had additional favourable effect in changes of PC cancer mortality, particularly in the youngest age groups. Further improvements in early detection methods followed by timely appropriate treatment are essential for decreasing mortality from BC and PC. Future studies and data on risk factors, the use of mammography and PSA testing, the effectiveness of screening programmes and the causes of changes in BC mortality trends in the youngest generations in Lithuania are warranted.

## Supplementary Information

**Additional file 1.**

## Data Availability

The data that support the findings of this study are available in World Health Organisation database at [https://www.who.int/data/data-collection-tools/who-mortality-database], reference number [27] and Health Information Centre of the Institute of Hygiene, Lithuania at [https://www.hi.lt/uploads/pdf/leidiniai/Statistikos/Mirties_priezastys/Mirties_priezastys_2020.pdf], reference number [28]. The data were also derived from the Statistics Lithuania: [https://osp.stat.gov.lt/statistiniu-rodikliu-analize#/], reference number [30].

## Abbreviations

ASMR: Age-standardised mortality rate
BC: Breast cancer
95% CI: 95% Confidence Interval
PC: Prostate cancer
PSA: Prostate-specific antigen
RRs: Rate ratios
WHO: World Health Organization
