# Supplementary material for: An analysis of time trends in breast and prostate cancer mortality rates in Lithuania, 1986–2020
Source: BMC Public Health. 2022 Sep 23;22:1812. doi: 10.1186/s12889-022-14207-4 (PMC9508783; doi:10.1186/s12889-022-14207-4)
Supplement: Supplementary file 1 — Additional file 1. [file 12889_2022_14207_MOESM1_ESM.doc]

Table A. Rate ratios (95% CI) of periods and cohorts by cancer type.

|  | **Breast cancer** | | | **Prostate cancer** | | | |  | | |
| --- | --- | --- | --- | --- | --- | --- | --- | --- | --- | --- |
| Year of death, midpoint | |  | |  |  |  | | | | |
| 1988.5 | 1.05 (0.98; 1.13) |  | 0.68 (0.60; 0.76) | | | | |  | | |
| 1993.5 | 1.07 (1.01; 1.14) |  | 0.77 (0.71; 0.85) | | | | |  | | |
| 1998.5 | 1.06 (1.00; 1.12) |  | 0.85 (0.78; 0.92) | | | | |  | | |
| 2003.5 | 1.05 (0.99; 1.11) |  | 0.90 (0.84; 0.97) | | | | |  | | |
| 2008.5 | 1 |  | 1 | | | | |  | | |
| 2013.5 | 0.95 (0.90; 1.01) |  | 0.93 (0.87; 0.99) | | | | |  | | |
| 2018.5 | 0.93 (0.88; 0.98) |  | 0.89 (0.83; 0.96) | | | | |  | | |
| Year of birth, midpoint | |  |  | | | | |  |  | |
| 1901 | 0.44 (0.34; 0.56) |  | 0.34 (0.27; 0.43) | | | |  | | |  |
| 1906 | 0.51 (0.43; 0.60) |  | 0.44 (0.38; 0.51) | | | |  | | |  |
| 1911 | 0.72 (0.64; 0.80) |  | 0.53 (0.47; 0.61) | | | |  | | |  |
| 1916 | 0.78 (0.70; 0.86) |  | 0.66 (0.59; 0.75) | | | |  | | |  |
| 1921 | 0.96 (0.89; 1.05) |  | 0.81 (0.73; 0.90) | | | | |  | |  |
| 1926 | 0.98 (0.91; 1.05) |  | 0.84 (0.76; 0.92) | | | | |  | |  |
| 1931 | 1.04 (0.97; 1.11) |  | 0.88 (0.80; 0.97) | | | | |  | |  |
| 1936 | 0.97 (0.91; 1.03) |  | 0.84 (0.76; 0.93) | | | | |  | |  |
| 1941 | 1.01 (0.94; 1.07) |  | 0.90 (0.82; 0.99) | | | | |  | |  |
| 1946 | 1.00 |  | 1 | | | | |  | |  |
| 1951 | 0.96 (0.90; 1.03) |  | 1.03 (0.91; 1.17) | | | | |  | |  |
| 1956 | 0.86 (0.80; 0.93) |  | 1.04 (0.88; 1.23) | | | | |  | |  |
| 1961 | 0.71 (0.65; 0.77) |  | 0.93 (0.72; 1.35) | | | | |  | |  |
| 1966 | 0.58 (0.52; 0.65) |  | 0.84 (0.52; 1.35) | | | | |  | |  |
| 1971 | 0.58 (0.50; 0.66) |  | 0.52 (0.15; 1.79) | | | | |  | |  |
| 1976 | 0.51 (0.42; 0.62) |  |  | | | | |  | |  |
| 1981 | 0.60 (0.45; 0.80) |  |  | | | | |  | | |
| 1986 | 0.61 (0.39; 0.97) |  |  | | | | |  | | |
| 1991 | 0.89 (0.33; 2.38) |  |  | | | | |  | | |

^*^In all age-period-cohort analyses the reference group was the central calendar period and central birth cohort.

Table B. Statistical parameters for overall and age-specific annual percent changes in age-period-cohort models

| **Cancer type** | **Net drift (%/year, 95% CI)** | **P value** | | |
| --- | --- | --- | --- | --- |
|  |  | **All local drifts = net drift** | **All cohort deviations = 0** | **All period deviations = 0** |
| Breast | -0.48 (-0.70; -0.26) | < 0.001 | < 0.001 | 0.142 |
| Prostate | 0.96 (0.55; 1.37) | < 0.001 | < 0.001 | < 0.001 |
